# Survivin expression in ovarian cancer and its correlation with clinico-pathological, surgical and apoptosis-related parameters

**DOI:** 10.1038/sj.bjc.6602332

**Published:** 2005-01-18

**Authors:** G Ferrandina, F Legge, E Martinelli, F O Ranelletti, G F Zannoni, L Lauriola, M Gessi, V Gallotta, G Scambia

**Affiliations:** 1 Gynecologic Oncology Unit, Catholic University of the Sacred Heart, Largo A. Gemelli, 8, 00168 Rome, Italy; 2 Department of Histology, Catholic University of the Sacred Heart, Rome, Italy; 3 Department of Pathology, Catholic University of the Sacred Heart, Rome, Italy; 4 Department of Oncology, Catholic University of the Sacred Heart, Campobasso, Italy

**Keywords:** ovarian cancer, survivin, prognosis

## Abstract

We investigated the association of survivin expression with prognosis and other apoptosis-related biological factors in 110 primary ovarian cancer patients admitted to the Division of Gynecologic Oncology, Catholic University of Rome. Immunohistochemistry was performed on formalin-fixed, paraffin-embedded sections by using polyclonal antibody ab469 for survivin, and mouse monoclonal antibodies (clone 124 and DO-7), for bcl-2 and p53, respectively. Cytoplasmic survivin immunoreaction was observed in 84.5% cases, while nuclear survivin immunostaining was observed in 29.1% cases. We failed to find any relationship between cytoplasmic survivin positivity rate and any of the parameters examined. Serous tumours showed a lower percentage of nuclear survivin positivity with respect to other histotypes (20.5 *vs* 48.6%, respectively; *P*-value=0.004). The percentage of nuclear survivin positivity was higher in cases subjected to primary tumour cytoreduction (43.5%), with respect to patients subjected to exploratory laparotomy (20%) (*P*=0.024). Bcl-2 and p53 were, respectively, expressed in 27.3 and 60.0% of the cases and their expression was not correlated with survivin status. During the follow-up period, progression and death of disease were observed in 68 (61.8%) and 53 (48.2%) cases, respectively. There was no difference in time to progression and overall survival according to survivin status in ovarian cancer patients. In conclusion, in our experience, the immunohistochemical assessment of survivin status does not seem to be helpful in the prognostic characterisation of ovarian cancer. A more in depth investigation of the complex physiology of divergent survivin variants is needed in order to clarify the biological and the clinical role of differentially located survivin isoforms.

Although several clinicopathological parameters have been reported to be of prognostic significance in ovarian cancer, including F.I.G.O. stage, volume of residual disease, presence of cytologically malignant ascites and grade of tumour differentiation (Hoskins *et al*, 2000), it is conceivable that the assessment of biochemical factors more strictly related to tumour cell biology and intrinsic aggressiveness could help identifying high-risk patients and facilitating management of this disease.

Apoptosis (programmed cell death) has been proposed to play a role not only in cancer onset and progression but also in sustaining decreased tumour cell sensitivity to chemotherapy (Hickman, 1992; Thompson, 1995), which still represents one of the main prognostic indicators in this neoplasia (Hoskins *et al*, 2000). In this context, the analysis of the molecules possibly involved in the modulation of apoptosis seems to be particularly attractive.

Survivin is a member of the inhibitors of apoptosis protein (IAP) family, which participates in the complex network regulating programmed cell death and also cell division (Ambrosini *et al*, 1997; Altieri and Marchisio, 1999; Salvesen and Duckett, 2002). Survivin protein is commonly detected in fetal tissues but not in normal adult tissues, while being overexpressed in human cancer, thus suggesting the contribution of survivin gene reactivation in carcinogenesis (Ambrosini *et al*, 1997). The crucial role of survivin in protection from apoptosis is supported by the observations that forced expression of survivin counteracts cell death induced by several apoptotic stimuli (Li *et al*, 1998), while survivin inhibition by antisenses causes spontaneous apoptosis (Olie *et al*, 2000). Survivin activity seems to be mediated by the inhibition of caspase pathways and also interaction with the microtubules of the mitotic spindle during the G2/M phase of cell cycle (Deveraux and Reed, 1999; Li, 2003), which suggest that survivin could promote tumour growth by dual functions, that is, inhibition of apoptosis and induction of mitogenesis. Overexpression of survivin has been associated with parameters of aggressiveness and poor prognosis in several solid tumours, although conflicting data have also been reported (Li, 2003). As far as ovarian lesions are concerned, a higher percentage of survivin overexpression was found in borderline and malignant tumours with respect to benign lesions (Sui *et al*, 2002).

Several studies have attempted to delineate the clinical role of survivin expression in epithelial ovarian carcinomas but no definitive conclusions could be drawn probably because of the size and/or heterogeneity of the population series, incompleteness of clinical informations as well as the use of different antibodies and methods of score of survivin expression (Yoshida *et al*, 2001; Sui *et al*, 2002; Takai *et al*, 2002; Zaffaroni *et al*, 2002; Cohen *et al*, 2003).

The aim of this study was to investigate the association of survivin expression with surgical data, response to chemotherapy and prognosis in a series of primary ovarian cancer patients undergoing surgery, chemotherapy treatment, and follow-up performed by the same gynaecological oncology team. Moreover, considering the biological relevance of the apoptosis regulatory proteins such as p53 and bcl-2 in the complex regulation of apoptosis (Miyashita and Reed, 1993; Miyashita *et al*, 1994), the association of survivin with these biological parameters has been also investigated.

## MATERIALS AND METHODS

### Patients

The study included 110 primary ovarian cancer patients admitted to the Division of Gynecologic Oncology, Catholic University of Rome between May 1986 and September 2002. The median age was 58.5 years (range 25–84). A total of 91 (82.7%) patients were stage III–IV. The other clinicopathological characteristics are listed in Table 1. According to the standard guidelines for ovarian cancer primary treatment, maximal surgical effort has been attempted in all patients resulting in successful debulking in 65 (59.1%) cases, which were subjected to surgical removal of tumour masses, along with total abdominal hysterectomy, adnexectomy, radical omentectomy, appendectomy and multiple biopsies, and additional surgery (intestinal resection, diaphragm stripping) when required. Radical pelvic and para-aortic lymphadenectomy was performed in all patients undergoing primary cytoreduction who had absent or microscopic residual disease or residual tumour less than 2 cm.

In total, 45 (40.9%) cases were judged to be unresectable at first surgery because of extensive peritoneal bulky carcinomatosis, agglutinated bowel/mesentery and infiltration of the upper gastrointestinal tract and/or the major vessels (Fanfani *et al*, 2003), and were submitted only to multiple biopsies.

Patients cytoreduced at first surgery received 4–6 cycles of chemotherapy 2–3 weeks after primary surgery, unless they showed clinical progression during treatment. As far as patients undergoing explorative laparotomy are concerned, they received three or four cycles of neoadjuvant chemotherapy before attempting a second cytoreductive surgery, unless they showed clinical progression during treatment. All patients underwent platinum-based chemotherapy (cisplatin: 75–100 mg m^−2^ for each cycle or carboplatin 5 AUC, q21), including also paclitaxel in 61 (58.6%) cases.

Response to chemotherapy was assessed by gynaecological exam, ultrasound examination, analysis of CA125 levels and CT scan, if necessary, and was recorded according to World Health Organization criteria (1979). In the subgroup of patients who were not susceptible to be cytoreduced at first surgery, a direct assessment of response to chemotherapy was carried out in case of clinical response, at the time of second laparotomy.

### Immunohistochemistry

Tumour tissues' biopsies from primary tumours were obtained at first surgery in all cases. Tissue specimens were fixed in formalin and paraffin-embedded according to standard procedures. In all, 3 *μ*m of representative blocks from each case were deparaffinised in xylene, rehydrated, treated with 3% H_2_O_2_ in TBS for 5 min to block endogenous peroxidase activity, and subjected to heat-induced epitope retrieval in microwave oven using 10 mM citric acid at pH 6.0. Sections were incubated with normal goat serum 20% for 30 min, then with polyclonal antibody ab496 (ABCAM Limited, Cambridge, UK) diluted according to the manufacturer's instructions, overnight. Bcl-2 and p53 were analysed using mouse monoclonal antibodies (clone 124 and DO-7, respectively, DAKO, Carpintera, CA, USA), according to the methods previously described (Ferrandina *et al*, 1999; Ferlini *et al*, 2003).

Slides from all cases studied were then simultaneously processed for immunohistochemistry using En Vision-rabbit+System-HRP DAKO (Carpintera, CA, USA). Diaminobenzidine was used as a chromogen (DAB substrate System, DAKO). Negative controls were performed using nonimmunised rabbit serum or by omitting the primary antiserum.

The analysis of all tissue sections was carried out without any prior knowledge of the clinical parameters by three authors (MG, LL, FL) by means of light microscopy. Proportion of immunostained cells was scored at low magnification (× 5 objective lens) by evaluating the entire tumour area. The intensity of staining (scale 0–4) and the percentage of stained cells were evaluated. The following cutoff were ‘*a priori*’ chosen for scoring: cases with more than 20% of cells showing intensity of cytoplasmic staining >1 were considered positive for cytoplasmic survivin expression, while cases with nuclear staining ⩾1 in more than 5% of cells were considered as nuclear survivin positive. In case of disagreement (*n*=13, 11.8%), sections were submitted to a rejoint evaluation.

### Statistical analysis

Fisher's exact test or *χ*^2^ test were used to analyse the distribution of surviving-positive cases according to clinicopathological, surgical, and biological features.

Overall survival (OS) and time to progression (TTP) were calculated from the date of diagnosis to the date of death/progression or date last seen. Medians and life tables were computed using the product-limit estimate by the Kaplan and Meier method (1958), and the log-rank test was employed to assess the statistical significance (Mantel, 1966). Statistical analysis was carried out using SOLO (BMDP Statistical Software, Los Angeles, CA, USA).

## RESULTS

### Survivin immunostaining

**Figure 1:**
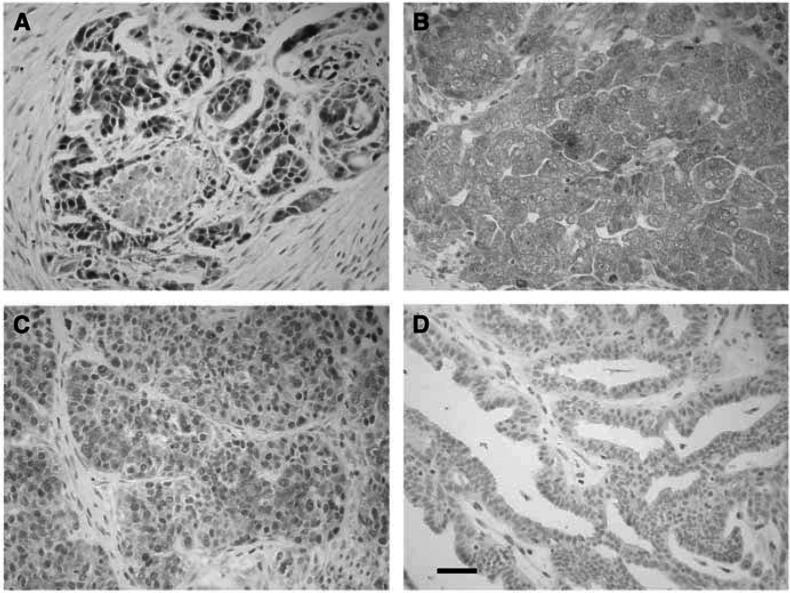
Survivin immunostaining in primary ovarian cancer. Specific survivin immunostaining was observed in both the cytoplasm and the nuclear compartment (**A**), either in the cytoplasm only (**B**), or in nuclear compartment only (**C**). An example of surviving-negative ovarian tumour (**D**). Bar=35 *μ*m.

As shown in Figure 1, specific survivin immunostaining was observed both in the cytoplasm and nuclear compartment (A) of tumour cells, or only in the cytoplasm (B) or nucleus (C).

Cytoplasmic survivin immunoreaction was observed in 93 (84.5%) cases, while nuclear survivin immunostaining was observed in only 32 (29.1%) cases: 29 (26.4%) cases showed survivin staining both in nuclear and cytoplasmic compartment.

There seems to be no association between cytoplasmic and nuclear survivin staining; however, nine out of 13 (69.2%) cases with very strong cytoplasmatic intensity (score=4) showed also nuclear staining in contrast with only 19 out of 80 (23.7%) cases with lighter (score=2,3) cytoplasmic staining (*P*=0.002).

### Correlation with clinical, surgical and pathological parameters

Table 2 shows the distribution of cytoplasmic and nuclear survivin positivity according to clinicopathological characteristics. As far as cytoplasmic survivin reaction is concerned, we failed to find any relationship between positivity rate and any of the clinicopathological parameters examined. As far as nuclear survivin expression is concerned, serous tumours showed a lower percentage of nuclear survivin positive immunoreaction with respect to other histotypes (20.5 *vs* 48.6%, respectively; *P*-value=0.004). On the other hand, nuclear survivin staining was not differently distributed according to other clinical–pathological parameters. No association between survivin status and response to chemotherapy was found. Moreover, neither nuclear nor cytoplasmic survivin staining were found to be associated with response to treatment in subgroups of patients receiving platinum-based *vs* paclitaxel-containing chemotherapy (data not shown).

Table 3 shows the distribution of cytoplasmic and nuclear survivin expression according to surgical features in stage III–IV ovarian cancer patients.

Cytoplasmic survivin immunostaining was not differently distributed according to any of the parameters analysed.

On the other hand, the percentage of nuclear survivin positivity was higher in cases in which tumour cytoreduction was achieved at primary surgery (20 out of 46, 43.5%) with respect to patients subjected to exploratory laparotomy (nine out of 45, 20%) (*P*=0.024). Nuclear survivin staining was found not to be different according to the extent of debulking since nuclear survivin positivity was found in seven out of 16 (43.7%) cases achieving optimal cytoreduction (residual tumour <0.5 cm) *vs* 13 out of 30 (43.3%) patients achieving suboptimal (residual tumour ⩾0.5 cm) debulking (*P*-value=n.s.).

We found a higher percentage of nuclear survivin positivity in patients with no apparent mesenteric infiltration (24 out of 60, 40.0%) with respect to patients with tumour mesenteric involvement (5 out of 31, 16.1%, *P*-value=0.017). In addition, the percentage of nuclear survivin positivity was also higher in cases without spread of disease in the upper region of the abdomen (52.2%) with respect to patients with this feature (24.3%, *P*-value=0.014).

### Correlation with apoptosis-related parameters

Bcl–2 and p53 expression was evaluated in 80 cases. Bcl-2 and p53 were expressed in 27.3 and 60.0%, respectively, of the cases examined. As shown in Table 4, neither cytoplasmic or nuclear survivin immunostaining were differently distributed according to any of the apoptosis-related parameters analysed.

### Survival analysis

Follow-up data were available for 110 patients. As of May 2004, the median follow up was 32 months (range 1–221). During the follow-up period, progression and death of disease were observed in 68 (61.8%) and 53 (48.2%) cases, respectively.

**Figure 2:**
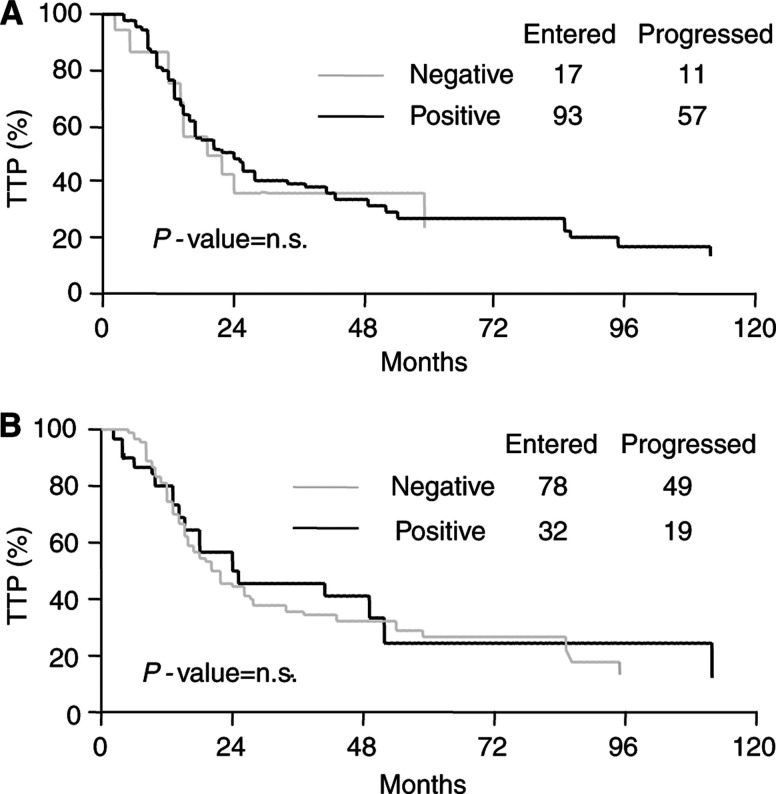
Time to progression (TTP) curves according to cytoplasmic (**A**) and nuclear (**B**) survivin status in ovarian cancer.

As shown in Figure 2, there was no difference in TTP according to cytoplasmic and nuclear survivin status in ovarian cancer patients. Similar results were observed when considering the OS curves (data not shown).

## DISCUSSION

Despite the availability of several studies exploring the expression of survivin protein in primary ovarian cancer, no definitive conclusions have been provided about its possible clinical role in this neoplasia (Yoshida *et al*, 2001; Sui *et al*, 2002; Takai *et al*, 2002; Zaffaroni *et al*, 2002; Cohen *et al*, 2003). As emphasised in Table 5, discrepancies among studies can be explained by size and clinical characteristics of the series examined (Yoshida *et al*, 2001; Sui *et al*, 2002; Takai *et al*, 2002; Cohen *et al*, 2003), as well as methodological differences: indeed, different antibodies as well as scoring systems for evaluation of survivin immunoreaction have been utilised. Moreover, survivin staining has been described as prevalent either in the cytoplasm (Yoshida *et al*, 2001; Takai *et al*, 2002; Zaffaroni *et al*, 2002) or in the nucleus (Sui *et al*, 2002; Takai *et al*, 2002; Cohen *et al*, 2003), and it has been not always clearly stated whether the overall rate of positivity was derived from a single subcellular compartment or both.

We first separately describe the expression of survivin protein in cytoplasmic and nuclear compartments of ovarian tumour cells. With the use of ab496 antibody, survivin was shown to be predominantly detected in the cytoplasmic compartment of ovarian cancer cells, confirming previous observations obtained with the same antibody after subcellular fractionation of HeLa cells (Fortugno *et al*, 2002). In particular, Fortugno *et al* (2002) showed that cytoplasmic and nuclear survivin pools are immunochemically different and this might partly explain the conflicting data on survivin localisation in solid tumours (Li, 2003). Besides, cytoplasmic and nuclear survivin are independently modulated during cell cycle progression and only cytoplasmic survivin associates with p34^cdc2^ and is phosphorylated on Thr34, event which seems to mediate the antiapoptotic function of the protein (O'Connor *et al*, 2000), thus suggesting that the two pools exert different biological functions. Moreover, data have been provided about the existence of functionally divergent survivin splice variants, which exhibit different subcellular localisations (Mahotka *et al*, 2002).

The relationship between immunohistochemically detected cytoplasmic and nuclear localisation of survivin has been not investigated: although we failed to find any relationship between cytoplasmic and nuclear staining, a higher percentage of cases with very strong cytoplasmic intensity showed also nuclear survivin immunoreaction. Further studies aimed at clarifying the functional relationship between cytoplasmic and nuclear survivin are needed in order to properly assess the role of immunohistochemically detected survivin pools in human cancer.

As far as the association between survivin and clinicopathological and surgical parameters is concerned, we showed that serous ovarian tumours expressed a significantly lower nuclear survivin content with respect to other histotypes, as also reported by Yoshida *et al* (2001). Moreover, an intriguing association between high expression of nuclear survivin and better chance of performing tumour cytoreduction at first surgery was shown, which is likely to be supported by the association of higher nuclear survivin content with the absence of distinctive patterns that usually preclude the feasibility of cytoreduction, such as tumour involvement of upper abdominal organs and mesentery (Fanfani *et al*, 2003). Our data are unlikely to be influenced by the initial tumour extension, since no correlation between survivin expression and stage of disease was found, as previously reported (Sui *et al*, 2002; Zaffaroni *et al*, 2002). However, the association between high nuclear survivin expression and better chance of cytoreduction, which represents one of the major determinants of response to chemotherapy and favourable prognosis (Hoskins *et al*, 2000), does not seem to translate into major differences in clinical outcome, perhaps because of the interference of other factors such as chemotherapy responsiveness or other yet unknown biological characteristics.

Our data seem to go against the original hypothesis that survivin might predict a more aggressive clinical outcome, although some evidences have been reported documenting the absence of any association between survivin expression and prognosis in some solid tumours (Grabowski *et al*, 2003; Li, 2003), and even a favourable prognostic role of high nuclear survivin content in gastric and bladder tumours (Okada *et al*, 2001; Lehner *et al*, 2002). The possibility that the biological and clinical role of survivin expression might also be influenced by tissue specificity cannot be ruled out and deserves further attention.

*In vitro* evidences showed that survivin might counteract chemotherapy-induced apoptosis (Zaffaroni *et al*, 2002), although univocal data have not been reported (Grossman *et al*, 2001; Pennati *et al*, 2002), possibly because of the use of different cell systems or the occurrence of peculiar surviving–cytotoxic drug interactions: for instance, survivin is able to bind polymerised microtubules through a putative tubulin-binding domain in the extended survivin C-terminal *α*-helix (Verdecia *et al*, 2000), and counteract paclitaxel-induced apoptosis in NIH3T3 fibroblasts (Li *et al*, 1998). In clinical studies, Zaffaroni *et al* (2002) showed that high levels of survivin protein are associated with resistance to regimens containing the microtubule-targeting agent paclitaxel, but are unrelated to cisplatin responsiveness in advanced ovarian cancer. However, no data on the clinical relevance of this finding in terms of patient clinical outcome have been provided (Zaffaroni *et al*, 2002).

In the current series, which included patients with measurable disease at first surgery in order to give a better evaluation of chemotherapy response, we failed to find any association between cytoplasmic or nuclear survivin expression and response to chemotherapy, even after subgrouping patients administered platinum-based *vs* paclitaxel-containing regimens. In addition, we could not find any difference in terms of TTP and OS according to either cytoplasmic or nuclear survivin status, in contrast with earlier studies which reported a negative prognostic role of survivin overexpression (Yoshida *et al*, 2001; Sui *et al*, 2002; Takai *et al*, 2002). However, it has to be taken into account that this is the first study examining the clinical relevance of survivin status in terms of clinical outcome in a single-institution large series of ovarian carcinomas, compared to previous reports which referred to very small sample series (Yoshida *et al*, 2001; Sui *et al*, 2002; Takai *et al*, 2002), sometimes biased by the inclusion of selected groups of patients (Yoshida *et al*, 2001).

Finally, the association between survivin, p53 and bcl-2 protein has been investigated based on the following background: (i) in the complex regulation of apoptosis and cell cycle progression, p53 and bcl-2 play a crucial role (Miyashita and Reed, 1993; Miyashita *et al*, 1994); (ii) wild-type p53 has been shown to negatively regulate human survivin at both mRNA and protein levels in 2774 ovarian carcinoma cells (Mirza *et al*, 2002), and to suppress survivin expression in lung adenocarcinoma cells (Hoffman *et al*, 2002); (iii) survivin expression has been associated with mutant p53 accumulation in ovarian and gastric cancer (Lu *et al*, 1998; Cohen *et al*, 2003), and during colorectal carcinogenesis (Kawasaki *et al*, 2001); moreover, a coassociation of survivin and bcl-2 has been found in breast and gastric cancer (Tanaka *et al*, 2000; Kawasaki *et al*, 2001). Our study, as well as other reports (Zaffaroni *et al*, 2002; Cohen *et al*, 2003), failed to show any relationship between p53 or bcl-2 and survivin expression, suggesting that these proteins could exert their functions through different mechanisms.

In conclusion, in our experience on a large series of patients, the immunohistochemical assessment of cytoplasmic and nuclear survivin status does not seem to be helpful in the prognostic characterisation of ovarian cancer. However, a more in depth investigation of the complex physiology of divergent survivin variants is needed in order to clarify the biological and possibly the clinical role of differentially located survivin isoforms.

Moreover, it is conceivable that, even though survivin has no prognostic role in ovarian cancer, it might be a potential target for apoptosis-based therapy, as testified by the increasing number of approaches aimed at (i) blocking survivin in cancer cells by small molecule antagonists, antisense oligonucleotides, ribozymes, dominant negative mutants (Reed and Wilson, 2003) or (ii) utilising survivin to create a tumour vaccine with dendritic cells (Pisarev *et al*, 2003; Reed and Wilson, 2003).

## Figures and Tables

**Table 1:** Patient characteristics

**Characteristics**	**No. of patients (%)**
All cases	110
Age (years)	
<60	56 (50.9)
⩾ 60	54 (49.1)

Ascites	
No	47 (42.7)
Yes	63 (57.3)

Histotype	
Serous	73 (66.4)
Mucinous	4 (3.6)
Endometrioid	21 (19.1)
Undifferentiated	7 (6.4)
Clear cell	3 (2.7)
n.a.	2 (1.8)

Grade	
G1–2	26 (23.4)
G3	74 (67.3)
n.a.	10 (9.3)
	
FIGO stage	
I–II	19 (17.3)
III–IV	91 (82.7)

Response to chemotherapy^a^	
Complete/partial	68 (74.7)
No change/progression	23 (25.3)

a Only FIGO stage III–IV.

**Table 2:** Nuclear and cytoplasmic survivin expression according to clinical and pathological characteristics in ovarian cancer

**Characteristics**	**No. of patients**	**Cytoplasmic survivin positive No. (%)**	***P*-value^a^**	**Nuclear survivin positive No. (%)**	***P*-value^a^**
All cases	110	93 (84.5)		32 (29.1)	—
Age (years)					
<60	56	47 (83.9)		18 (32.1)	
⩾60	54	46 (85.2)	0.9	14 (25.9)	NS

Ascites					
No	47	39 (82.9)		14 (30.4)	
Yes	63	54 (85.7)	0.4	18 (30.0)	0.3
				—	
Histotype					
Serous	73	62 (84.9)		15 (20.5)	
Mucinous	4	3 (75.0)		2 (50.0)	
Endometrioid	21	19 (90.5)		8 (38.1)	
Undifferentiated	7	6 (85.7)		5 (71.4)	
Clear cell	3	1 (33.3)	0.9^b^	2 (66.7)	**0.004** b
n.a.	2	—			

Grade					
G1–2	26	19 (73.0)		7 (26.9)	
G3	74	65 (87.8)		25 (33.8)	
n.a.	10	—	0.3	—	0.3

FIGO stage					
I–II	19	15 (78.9)		3 (15.8)	
III–IV	91	78 (85.7)	0.5	29 (31.9)	0.2

Response to chemotherapy^c^					
Complete/partial	68	59 (86.8)		20 (29.4)	
No change/progression	23	19 (82.6)	0.4	8 (34.8)	0.4

a Calculated by Fisher's exact test for proportion.

b Serous *vs* other histotypes.

c Only FIGO stage III–IV.

NS=not significant. Bold indicates significant *P*-values.

**Table 3:** Nuclear and cytoplasmic survivin expression according to surgical parameters in stage III–IV ovarian cancer

**Characteristics**	**No. of patients**	**Cytoplasmic survivin positive No. (%)**	***P*-value^a^**	**Nuclear survivin positive No. (%)**	***P*-value^a^**
All cases	91	78 (85.7)		29 (31.9)	—
Surgery					
Cytoreduction	46	39 (84.8)		20 (43.5)	
Explorative laparotomy	45	39 (86.7)	0.9	9 (20.0)	**0.024**

Carcinomatosis					
No	25	19 (76.0)		10 (40.0)	
Yes	66	59 (89.4)	0.1	19 (28.8)	0.20

Mesenteric infiltration					
No	60	52 (86.7)		24 (40.0)	
Yes	31	26 (83.9)	0.5	5 (16.1)	**0.017**

Frozen pelvis					
No	61	52 (85.2)		18 (29.5)	
Yes	30	26 (86.7)	0.6	11 (36.7)	0.3

High diaphragmatic spread					
No					
Yes	23	19 (82.6)		12 (52.2)	
	70	59 (84.3)	0.5	17 (24.3)	**0.014**

a Calculated by Fisher's exact test for proportion. Bold indicates significant *P*-values.

**Table 4:** Cytoplasmic and nuclear survivin expression according to apoptosis-related parameters in ovarian cancer

**Characteristics**	**No. of patients**	**Cytoplasmic survivin positive No. (%)**	***P*-value^a^**	**Nuclear survivin positive No. (%)**	***P*-value^a^**
Bcl-2 status					
Negative	58	48 (82.7)		23 (9.6)	
Positive	22	17 (77.3)	NS	7 (31.8)	NS

P53 status					
Negative	32	28 (87.5)		9 (28.1)	
Positive	48	41 (85.4)	NS	14 (29.2)	NS

a Calculated by Fisher's exact test for proportion.

NS=not significant.

**Table 5:** Studies examining the expression and clinical role of survivin in ovarian cancer

								**Relation with**		**Correlation with poor survival**		**Correlation with poor response to chemotherapy**	
**Author (year)**	**Prevalent expression in**	**No.**	**Method (section)**	**Antibody**	**Positivity expressed on**	**Cutoff**	**% positive cases**	**p53**	**bcl-2**	**Uni**	**Multi**	**Uni**	**Multi**
Yoshida (2001)	Cytoplasm	32^a^	IC Paraffin	Surv11A	ND	Weighted score >5^b^	18.7	—	—	Yes	—	—	—
Takai (2002)	Nucleus	26	IC Cryostat	FL-142	ND	>60% positive cells	34.6	—	—	Yes	—	—	—
Sui (2002)	Nucleus	47	IC Paraffin	Surv11A	ND	>50% positive cells	51.1	—	—	Yes	—	—	—
Zaffaroni (2002)	Cytoplasm	124	IC Paraffin	Ab469	Cytoplasm and/or Nucleus	>30% positive cells	72.5	No	—	—	—	Direct	Direct
Cohen (2003)	Nucleus	49	IC Paraffin	FL-142	Cytoplasm and/or Nucleus	ND	74.0	Yes	No	No	—	—	—
Current study	Cytoplasm	110	IC Paraffin	Ab469	Cytoplasm	>20% cells (score>1)	84.5	No	No	No	—	No	—
					Nucleus	>5% cells (score⩾1)	29.1	No	No	No	—	No	—

Uni=univariate analysis; Multi=multivariate analysis; IC=immunohistochemistry; ND=not defined.

a Including 16 clear cell and 16 serous adenocarcinomas.

b Weighted score obtained by multiplying the percentage of positive cells and intensity of staining.
